# 3D Differentiation of Bone‐Marrow Derived Mesenchymal Stromal Cells into the Keratocyte Lineage for Corneal Bioprinting

**DOI:** 10.1002/adhm.202405073

**Published:** 2025-07-29

**Authors:** Alexandre Taoum, Ronja Friede, Ole Thaden, Oleksandr Rachynskyi, Andrea Frank, Meng Wang, Friederike Dehli, Matthias Fuest, Daniela Duarte Campos

**Affiliations:** ^1^ Bioprinting & Tissue Engineering Group Center for Molecular Biology of Heidelberg University; ^2^ Ophthalmology Clinic RWTH Aachen University Hospital

**Keywords:** corneal opacity, corneal scarring, corneal bioprinting, differentiation, keratocyte

## Abstract

Corneal stromal keratocytes (CSK) play a critical role in maintaining corneal transparency and biomechanical integrity. However, challenges in obtaining enough functional CSK limit the progress of corneal tissue engineering and bioprinting. This study presents a novel protocol for the differentiation of bone marrow‐derived mesenchymal stromal cells (BM‐MSC) into CSK‐like cells, optimized for both 2D and 3D culture systems, including 3D bioprinted constructs. A key innovation of this work is the preconditioning of BM‐MSC on collagen‐coated plates to reduce α‐SMA expression, a marker associated with myofibroblast differentiation and fibrosis. Immunofluorescence and qPCR analyses demonstrated significant upregulation of CSK‐specific markers, including Lumican, Keratocan, ALDH1A1, ALDH3A1, and Collagen I, while α‐SMA expression remained undetectable. Notably, 3D cultures provided an enhanced environment for CSK differentiation, mimicking the corneal stroma more closely than 2D cell culture systems. The combination of preconditioning and 3D culture systems offers a promising approach for the biofabrication of functional corneal stroma, paving the way for future therapies aimed at treating corneal opacity and scarring.

## Graphical abstract

This study introduces a novel differentiation protocol for generating corneal stromal keratocyte‐like cells using drop‐on‐demand bioprinting and a collagen‐based hydrogel. The bioprinted constructs maintained a stable dome shape over 21 days while exhibiting upregulated CSK markers postdifferentiation. This work advances biofabrication methods for corneal tissue engineering and personalized regenerative medicine applications.

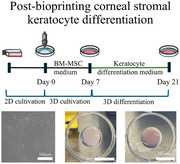

## 1 Introduction

The human cornea plays a central role in visual health and sight acuity due to its refractive function.^[^ ^1^ ^]^ Preserving corneal integrity is crucial, as corneal diseases and injuries continue to be one principal cause of blindness worldwide, affecting millions of individuals.^[^ ^2^ ^]^ Corneal grafting, first performed ≈120 years ago, has become a standard surgical procedure that has continuously evolved over the decades and is widely documented and protocolized.^[^ ^3^ ^]^ Nevertheless, drawbacks remain, mainly the donor's shortage,^[^ ^4^ ^]^ the risk of graft rejection,^[^ ^5^ ^]^ and post‐keratoplasty infections.^[^ ^6^ ^]^ Corneal tissue engineering has emerged as a promising field in regenerative medicine, especially for corneal transplants, affected by donor shortage.^[^ ^7^ ^]^ 3D bioprinting based on constructing structures layer by layer using hydrogels coupled with cells, offers an emerging approach to fabricate patient‐specific corneal tissues.^[^ ^8^ ^]^

Representing ≈90% of the corneal thickness, the stroma stands out as its predominant layer. It consists of highly organized collagen fibers, mainly type I, arranged in lamellae. The stroma is mostly composed of one cell type, embedded between these collagen fibers, called corneal stromal keratocytes (CSK), responsible for maintaining the extracellular matrix.^[^ ^9^ ^]^ Duarte Campos et al. have shown that CSK can be printed in a collagen‐based bioink and kept their specific markers after 7 days in culture.^[^ ^10^ ^]^ However, the isolation of CSK presents significant challenges, characterized by the complexity and time‐consuming nature of the procedure, coupled with a low success rate.^[^ ^11^ ^]^

The use of differentiated Bone‐Marrow derived Mesenchymal Stromal Cells (BM‐MSC) as replacement of CSK is a promising approach in corneal tissue engineering. Although harvesting cells from the patient's own tissue for transplantation after sub‐culturing them is considered the optimal approach, the differentiation of BM‐MSC into the keratocyte lineage offers a hopeful alternative to corneal tissue engineering.^[^ ^12^ ^]^ Compared to CSK, the collection and isolation of BM‐MSC is easy and minimally invasive for the patient.^[^ ^13^ ^]^ Following isolation, BM‐MSC have good proliferation rates, and can be expanded in vitro for multiple population doublings,^[^ ^14^ ^]^ thereby guaranteeing a sustainable cell source for corneal tissue engineering.

Since the composition of the corneal stroma primarily consists of type I collagen, employing collagen emerges as the optimal choice for encapsulating cells prior to their differentiation. A bioink formulated with type I collagen would represent an optimal choice for corneal bioprinting, nevertheless, collagen utilization is known in the bioprinting community as being notoriously challenging to print due to its low mechanical properties.^[^ ^15^, ^16^ ^]^ Moreover, following its crosslinking, lab‐grade collagens become opaque due to their concentrations,^[^ ^17^ ^]^ excluding their use as the only matrix for tissue‐engineered cornea substitutes. Our previous studies have shown that an agarose‐collagen blend is a suitable candidate for corneal tissue engineering,^[^ ^10^ ^]^ and this bioink was shown to be suitable for BM‐MSC drop‐on‐demand bioprinting, with a viability rate above 90%.^[^ ^18^ ^]^

Recent advancements have demonstrated the potential of BM‐MSC to differentiate into various cell types, including CSK‐like cells, under specific culture conditions.^[^ ^19^ ^]^ However, existing differentiation protocols often face challenges in maintaining the functional phenotype of CSK cells, particularly in avoiding the unintended differentiation into myofibroblasts. Myofibroblast differentiation is undesirable as they induce fibrotic changes that can impair corneal transparency and biomechanical properties.^[^ ^20^ ^]^ Consequently, there is a critical need for optimized differentiation strategies that promote CSK marker expression while suppressing myofibroblast formation.

In this context, the present study introduces a novel differentiation protocol designed to generate CSK‐like cells from BM‐MSC using both 2D and 3D culture systems. This protocol emphasizes the preconditioning of BM‐MSC on collagen‐coated plates prior to differentiation, a strategy aimed at reducing the expression of the myofibroblast marker α‐smooth muscle actin (α‐SMA). Additionally, the use of a 3D culture environment over a 21‐day period provides a physiologically relevant setting that enhances the expression of key CSK markers, such as Lumican, Keratocan, ALDH1A1, and ALDH3A1.^[^ ^11^ ^]^ By closely mimicking the native corneal microenvironment, the 3D culture system facilitates the generation of CSK‐like cells with enhanced functionality and reduced fibrotic potential. Moreover, our 3D differentiation protocol was successfully implemented on 3D bioprinted constructs, enabling the generation of CSK‐like cells with stable phenotypic and structural properties after 21 days of culture.

## 2 Results

### 2.1 Immunofluorescence Staining in 2D Culture and Differentiation

The expression of primary CSK markers in BM‐MSC before and after differentiation was assessed by immunofluorescence staining. The primary markers analyzed included Lumican, Keratocan, ALDH1A1, ALDH3A1, and Collagen I, together with the negative marker α‐SMA. IF figures show only one biological replicate per condition, the two other donors can be found in the supporting information. HSF was used as positive control for the α‐SMA staining (Figure S9, Supporting Information).

### 2.2 Pre‐Differentiation Analysis

**Figure 1:**
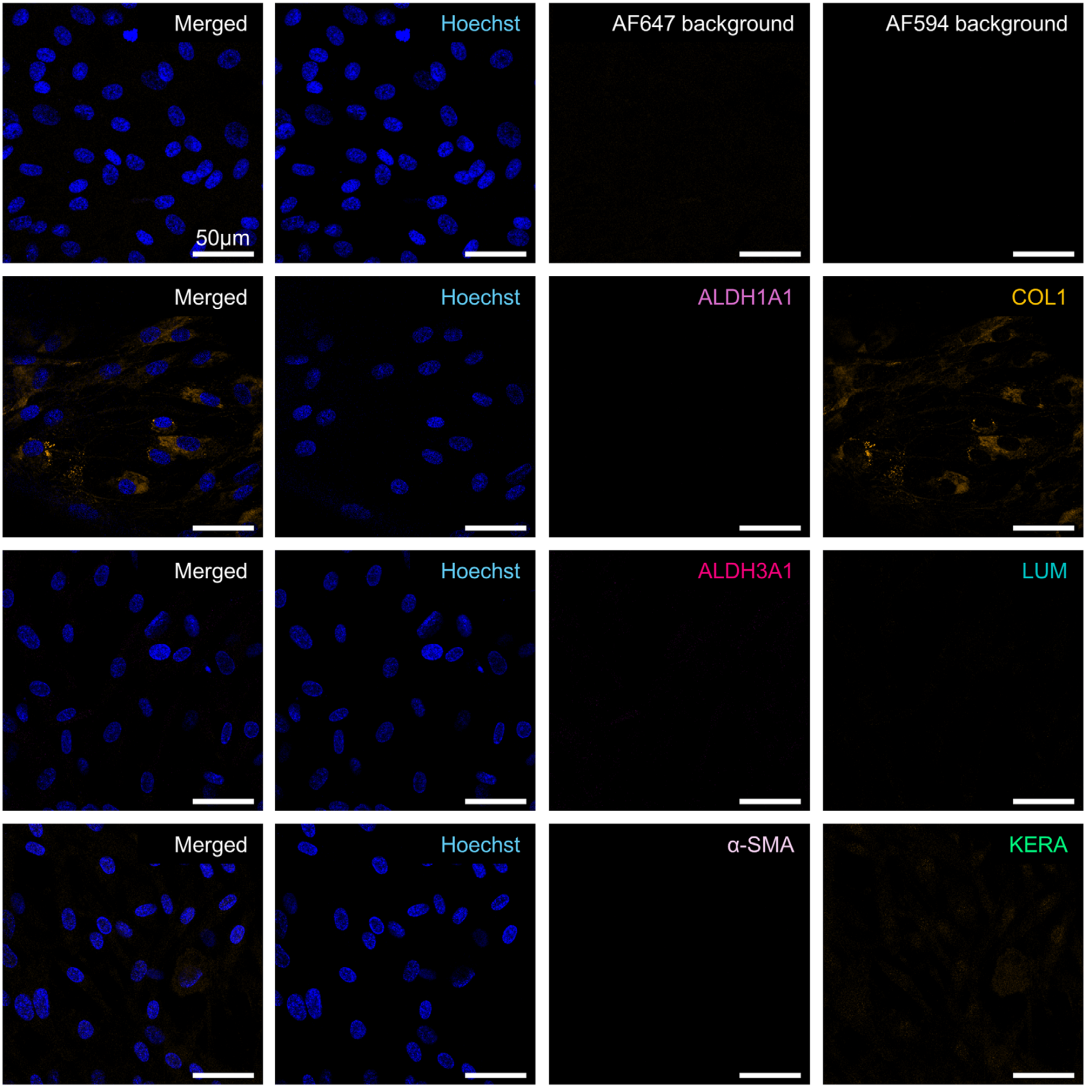
Immunofluorescence staining of 2D BM‐MSC for ALDH1A1 (light pink), Collagen 1 (COL1, orange), ALDH3A1 (dark pink), Lumican (LUM, turquoise), α‐SMA (lilac), and Keratocan (KERA, green) with Hoechst Nuclear Staining (blue) and Background Controls prior to differentiation. Scale bars represent 50 µm. Representative images from samples prepared with donor Male 63. Images from donors Females 72 and 62 are in the supporting information.

In the undifferentiated BM‐MSC, the expression of the CSK‐specific markers Lumican, Keratocan, ALDH1A1, and ALDH3A1 was absent, as indicated by non‐detectable fluorescence signals. This absence confirms the undifferentiated state of the BM‐MSC and their non‐CSK identity at this stage. A minimal fluorescence signal was detected for Collagen I, suggesting a low basal expression of this extracellular matrix protein in the undifferentiated BM‐MSC. Additionally, no signal for α‐SMA was observed (**Figures** **1**; S1 and S2, Supporting Information).

### 2.3 Post‐Differentiation Analysis

**Figure 2:**
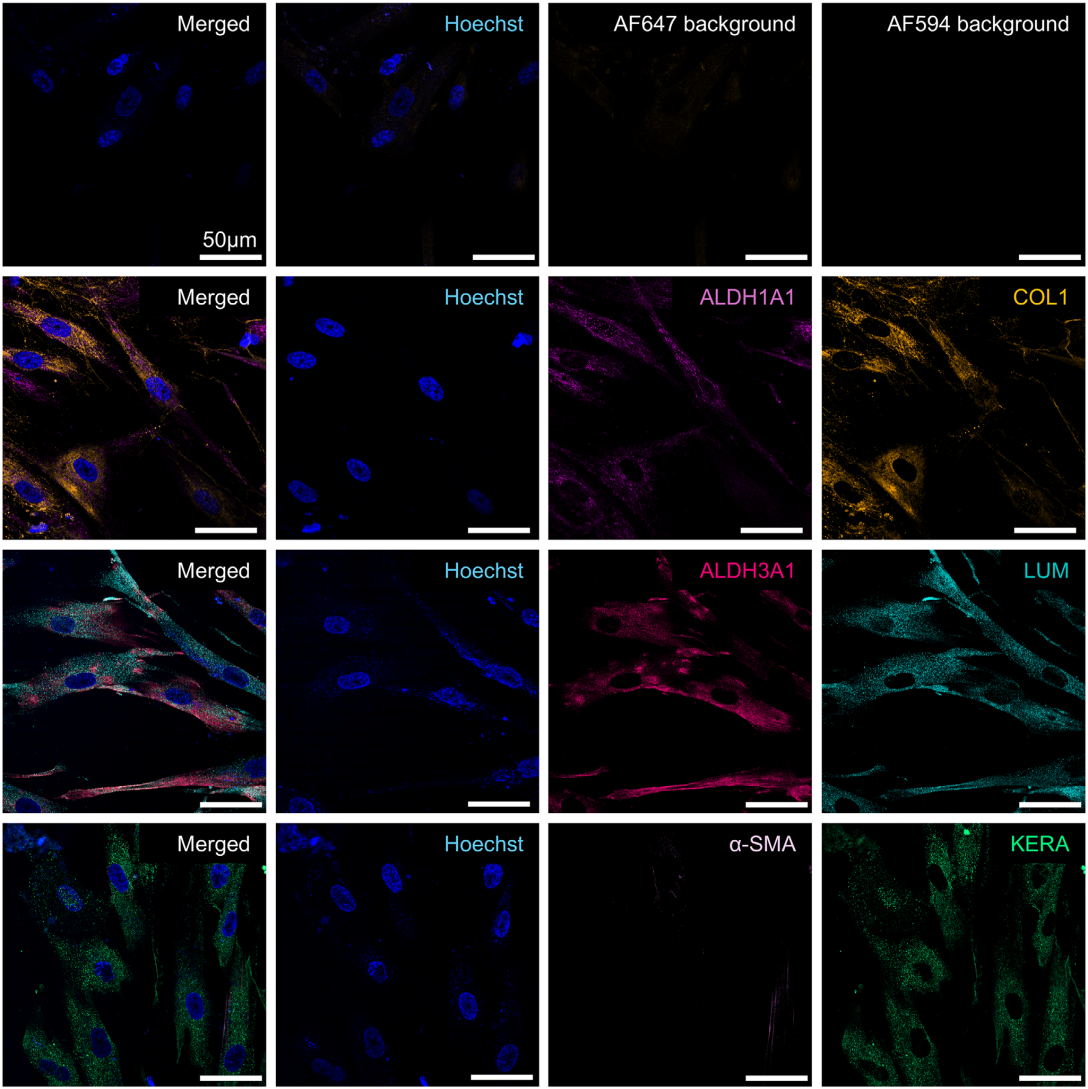
Immunofluorescence staining of 2D CSK‐MSC for ALDH1A1 (light pink), Collagen 1 (COL1, orange), ALDH3A1 (dark pink), Lumican (LUM, turquoise), α‐SMA (lilac), and Keratocan (KERA, green) with Hoechst Nuclear Staining (blue) and Background Controls post‐differentiation. Scale bars represent 50 µm. Representative images from samples prepared with donor Male 63. Images from donors Females 72 and 62 are in the supporting information.

After 14 days of differentiation, an upregulation in the expression of CSK‐specific markers was observed. Strong fluorescence signals for Lumican, Keratocan, ALDH1A1, and ALDH3A1 were evident, confirming successful differentiation of BM‐MSC into cells expressing CSK phenotypic markers. The expression of Collagen I was also enhanced, indicating the establishment of a corneal stromal extracellular matrix environment. Importantly, α‐SMA remained undetectable post‐differentiation, which is a favorable outcome as the absence of α‐SMA aligns with the non‐myofibroblastic phenotype typical of CSK, and suggests the avoidance of fibrosis or scarring pathways during differentiation (**Figures** **2**; S3 and S4, Supporting Information).

These findings show that the differentiation protocol effectively induced the BM‐MSC to adopt a CSK‐like phenotype, as evidenced by the expression of primary CSK markers and the continued absence of the myofibroblast marker α‐SMA.

### 2.4 Immunofluorescence Staining in 3D Culture and Differentiation in Gels

The differentiation of BM‐MSC into CSK was further investigated in 3D hydrogel cultures that mimicked the corneal stroma extracellular matrix environment. The expression of key CSK markers, including Lumican, Keratocan, ALDH1A1, ALDH3A1, and Collagen I, was assessed via IF staining, with α‐SMA serving as a negative marker to monitor the absence of myofibroblast differentiation. IF figures show only one biological replicate per condition, the two other donors can be found in the supporting information.

### 2.5 Pre‐Differentiation Analysis in 3D Culture

**Figure 3:**
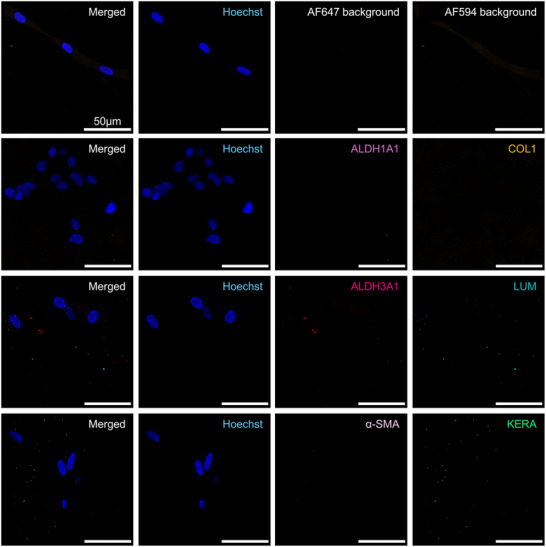
Immunofluorescence staining of 3D BM‐MSC for ALDH1A1 (light pink), Collagen 1 (COL1, orange), ALDH3A1 (dark pink), Lumican (LUM, turquoise), α‐SMA (lilac), and Keratocan (KERA, green) with Hoechst Nuclear Staining (blue) and Background Controls prior to differentiation. Scale bars represent 50 µm. Representative images from samples prepared with donor Female 62. Images from donors Female 72 and Male 63 are in the supporting information.

Before the initiation of differentiation, BM‐MSC encapsulated in 3D hydrogels exhibited no detectable expression of the primary CSK markers Lumican, Keratocan, ALDH1A1, and ALDH3A1, as indicated by the absence of corresponding fluorescence signals (**Figures** **3**; S5 and S6, Supporting Information). This absence confirms the undifferentiated state of the BM‐MSCs within the 3D environment. Like in 2D cultures, α‐SMA was not detected in 3D, indicating that the BM‐MSCs had not spontaneously differentiated into a myofibroblast phenotype.

### 2.6 Post‐Differentiation Analysis in 3D Culture

**Figure 4:**
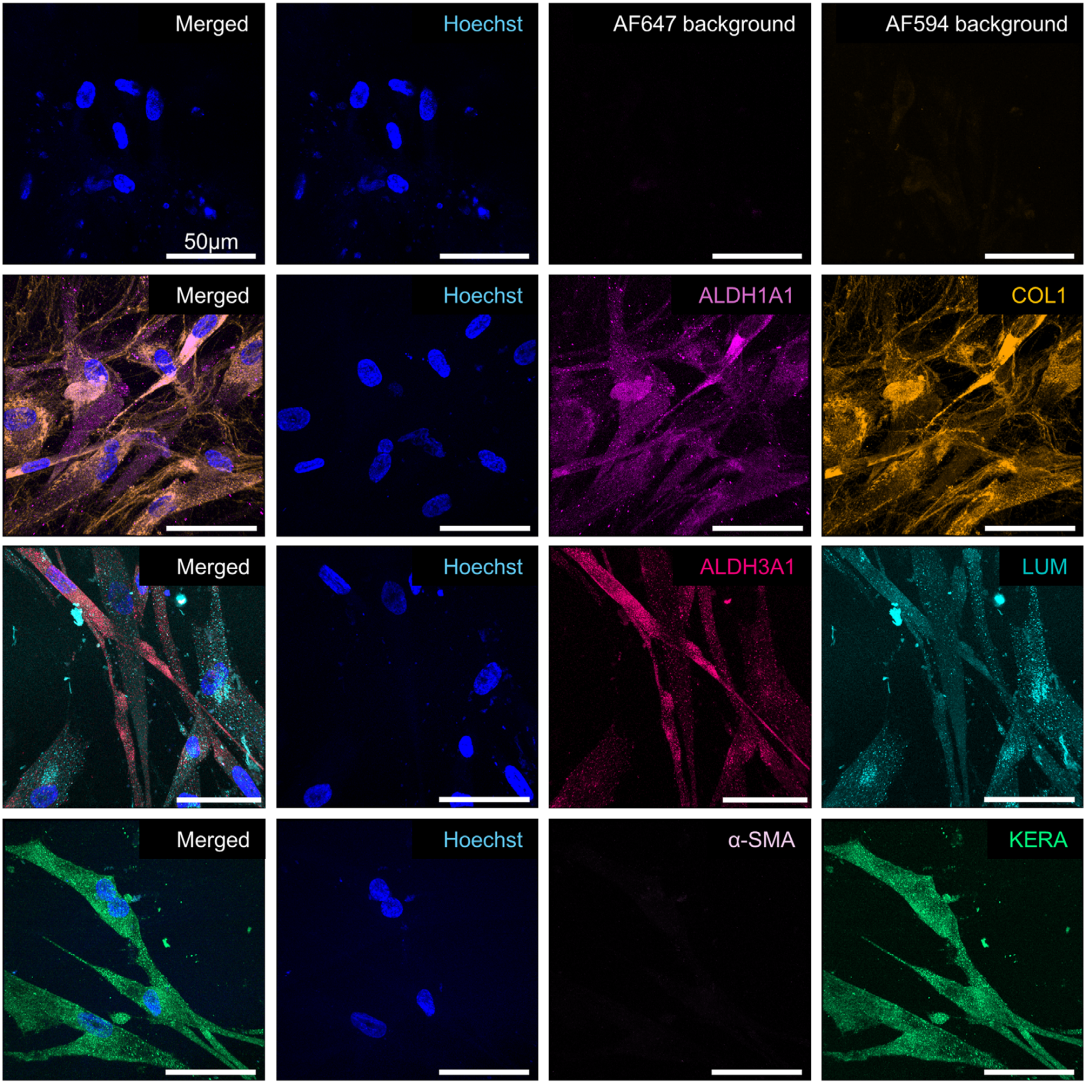
Immunofluorescence stainings of 3D CSK‐MSC for ALDH1A1 (light pink), Collagen 1 (COL1, orange), ALDH3A1 (dark pink), Lumican (Lum, turquoise), α‐SMA (lilac), and Keratocan (Kera, green) with Hoechst Nuclear Staining (blue) and Background Controls post‐differentiation. Scale bars represent 50 µm. Representative images from samples prepared with donor Female 62. Images from donors Female 72 and Male 63 are in the supporting information.

Following 21 days of culture and differentiation within the hydrogel matrix, induction of CSK marker expression was observed. Strong fluorescence signals were detected for Lumican, Keratocan, ALDH1A1, and ALDH3A1, confirming the successful differentiation of BM‐MSCs into a CSK‐like phenotype within the 3D culture system. The expression of Collagen I was also markedly enhanced, indicating an increased production of ECM components characteristic of the corneal stroma. Importantly, α‐SMA remained undetectable after differentiation, which is consistent with the non‐fibrotic, non‐myofibroblastic nature of primary CSK and indicates that the differentiation process in the 3D environment avoided pathways leading to fibrosis or scarring (**Figures** **4**; S7 and S8, Supporting Information).

These results underscore the efficacy of the 3D culture and differentiation system in promoting the expression of CSK‐specific markers while maintaining a phenotype without myofibroblast characteristics. The enhanced expression of stromal ECM components such as Collagen I further supports the potential of this 3D system for modeling corneal stroma and advancing tissue engineering applications.

### 2.7 Keratocyte Gene Expression Increased in 2D and 3D After Differentiation

**Figure 5:**
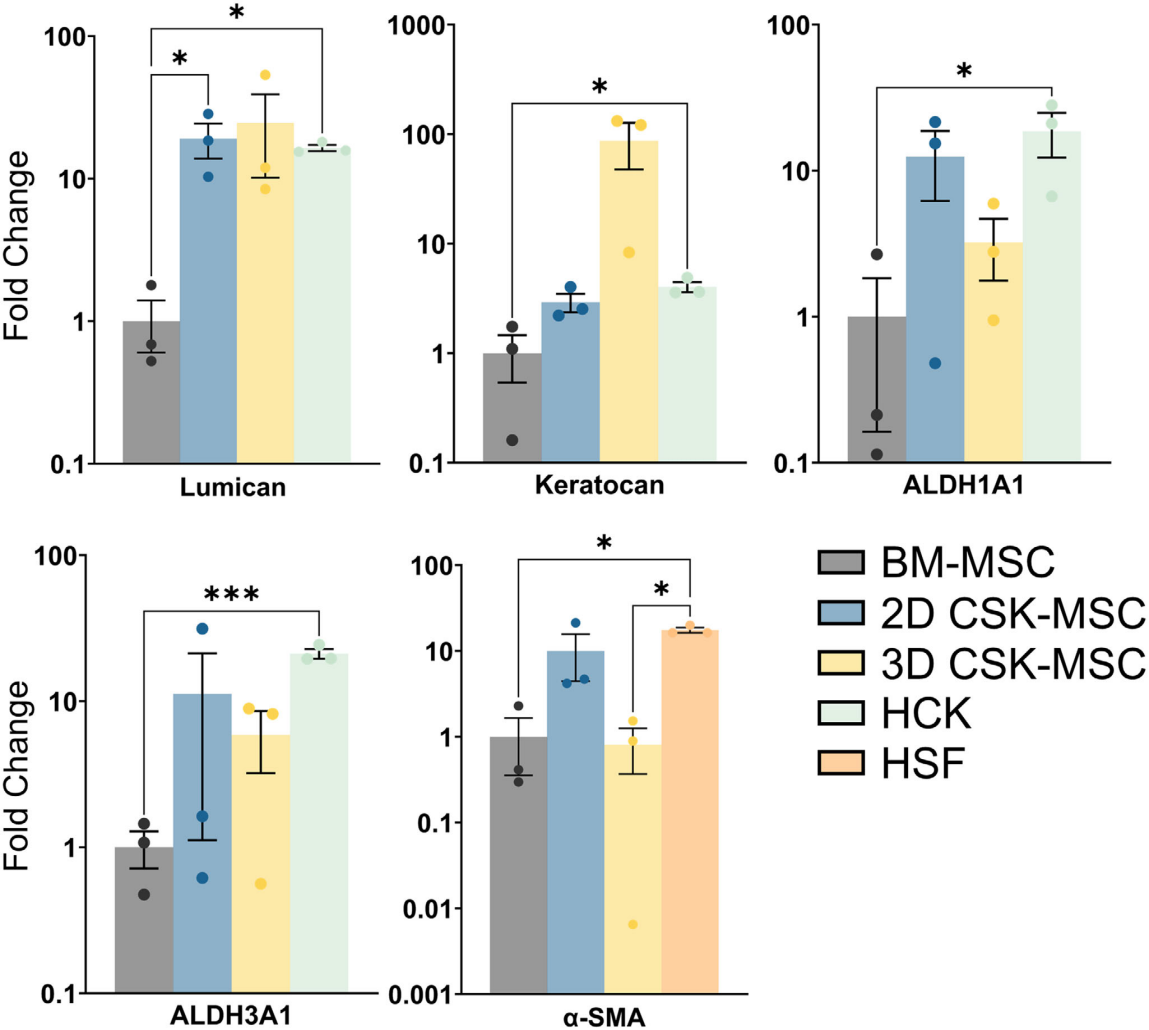
Comparative analysis of marker expression levels in BM‐MSC before and after differentiation in 2D and 3D cultures. Expression of CSK‐specific markers (Lumican, Keratocan, ALDH1A1, and ALDH3A1) and the myofibroblast marker (α‐SMA) in BM‐MSC before and after differentiation. Gene expression levels were normalized to GAPDH, and relative expression was calculated using the ΔΔCt method, with undifferentiated BM‐MSC set as the baseline (expression level = 1). HCK were used as positive controls for keratocyte markers, while HSF served as control for α‐SMA expression. (*p ≤ 0.05, ***p ≤ 0.001).

Comparing the relative gene expression of CSK markers from the BM‐MSC to the 2D and 3D differentiated cells and the primary HCK, the CSK marker Lumican was significantly upregulated for the 2D CSK‐MSC (22.09 ± 8.59, *p* = 0.014), upregulated for the 3D CSK‐MSC (28.46 ± 23.62, *p* = 0.818), and significantly highly expressed in the HCK (18.99 ± 1.35, *p* = 0.028) (**Figure** **5**). Keratocan was upregulated for the 2D CSK‐MSC (4.33 ± 1.17, *p* = 0.252), strongly upregulated for the 3D CSK‐MSC (129.26 ± 82.92, *p* = 0.012) and was significantly expressed in the HCK (5.98 ± 0.90, *p* = 0.011). ALDH1A1 was upregulated for both 2D CSK‐MSC (31.00 ± 21.99, *p* = 0.414) and 3D CSK‐MSC (8.02 ± 5.13, *p* = 0.568) and was significantly higher in the HCK (46.28 ± 22.18, *p* = 0.011). ALDH3A1 was upregulated in the 2D CSK‐MSC (12.39 ± 15.77, *p* = 0.486) and 3D CSK MSC (6.5 ± 4.18, *p* = 0.081) and significantly higher in the primary HCK (23.38 ± 2.51, *p* = 0.0005). No significant difference was found between 2D and 3D differentiated cells and primary HCK for all the primary CSK markers Lumican, Keratocan, ALDH1A1, and ALDH3A1. For the myofibroblast marker α‐SMA, 2D CSK‐MSC showed a non‐significant upregulation (15.33 ± 12.09, *p* = 0.160). Both BM‐MSC and 3D CSK‐MSC (1.23 ± 0.95, *p* = 0.0150) showed significantly lower α‐SMA expression compared to HSF (26.70 ± 2.60, *p* = 0.015).

### 2.8 Keratocyte Differentiation in a Bioprinted Corneal Model

**Figure 6:**
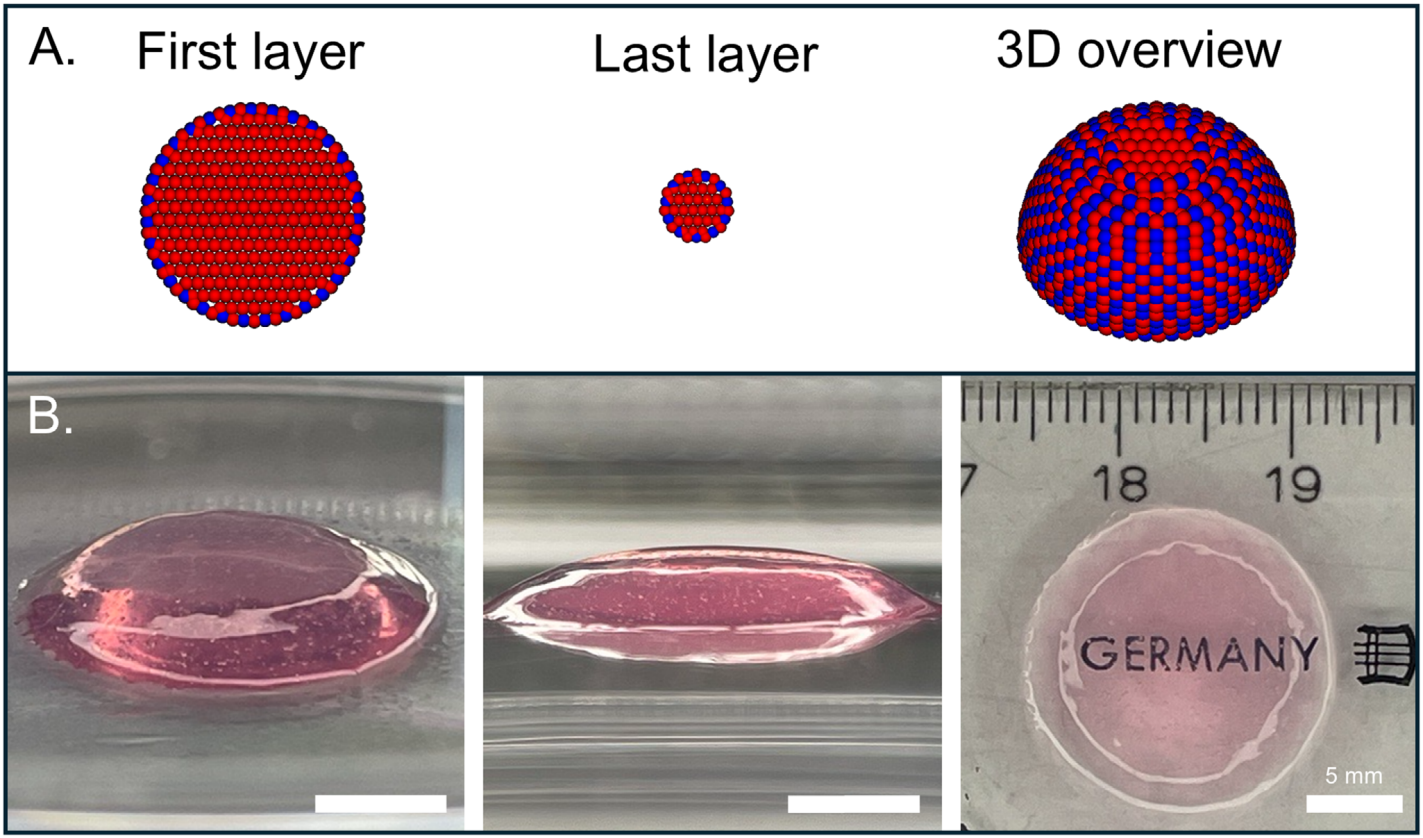
A) Schematic of the bottom‐up 3D model design. Dome‐shaped artificial cornea was designed by CAD and sliced: A (red) and B (blue) drops were bioprinted in an equally spaced deposition for each layer, then the filling was bioprinted. B) Printed corneal model with high transparency, demonstrating undistorted text beneath. Side views of the construct, highlighting the maintained curvature resembling native corneas. Scale bars represent 5 mm.

The 3D bioprinted construct was modeled as an upscaled half‐sphere to be adapted to our bioprinting method. Bioprinted 3D corneal models must meet certain optical properties, such as transparency and optical density comparable to native corneas. After 21 days in culture post‐printing, our corneal models maintained their shape and remained transparent, highlighting the stability and suitability of the construct for long‐term applications. Although the model was designed as a half‐sphere with an 18 mm height, the printed construct appeared flatter due to droplet spreading upon contact with the substrate in the absence of support material (**Figure** **6**B).

**Figure 7:**
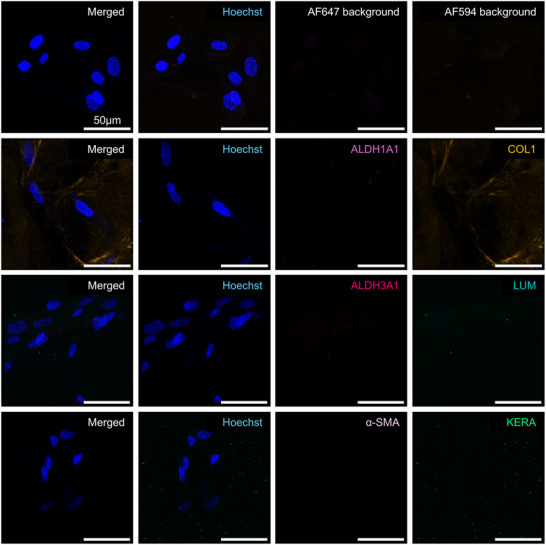
Immunofluorescence staining of 3D printed BM‐MSC for ALDH1A1 (light pink), Collagen1 (COL1, orange), ALDH3A1 (dark pink), Lumican (LUM, turquoise), α‐SMA (lilac), and Keratocan (KERA, green) with Hoechst Nuclear Staining (blue) and Background Controls prior to differentiation. Scale bars represent 50 µm. Representative images from samples prepared with pooled donors (*n* = 3).

**Figure 8:**
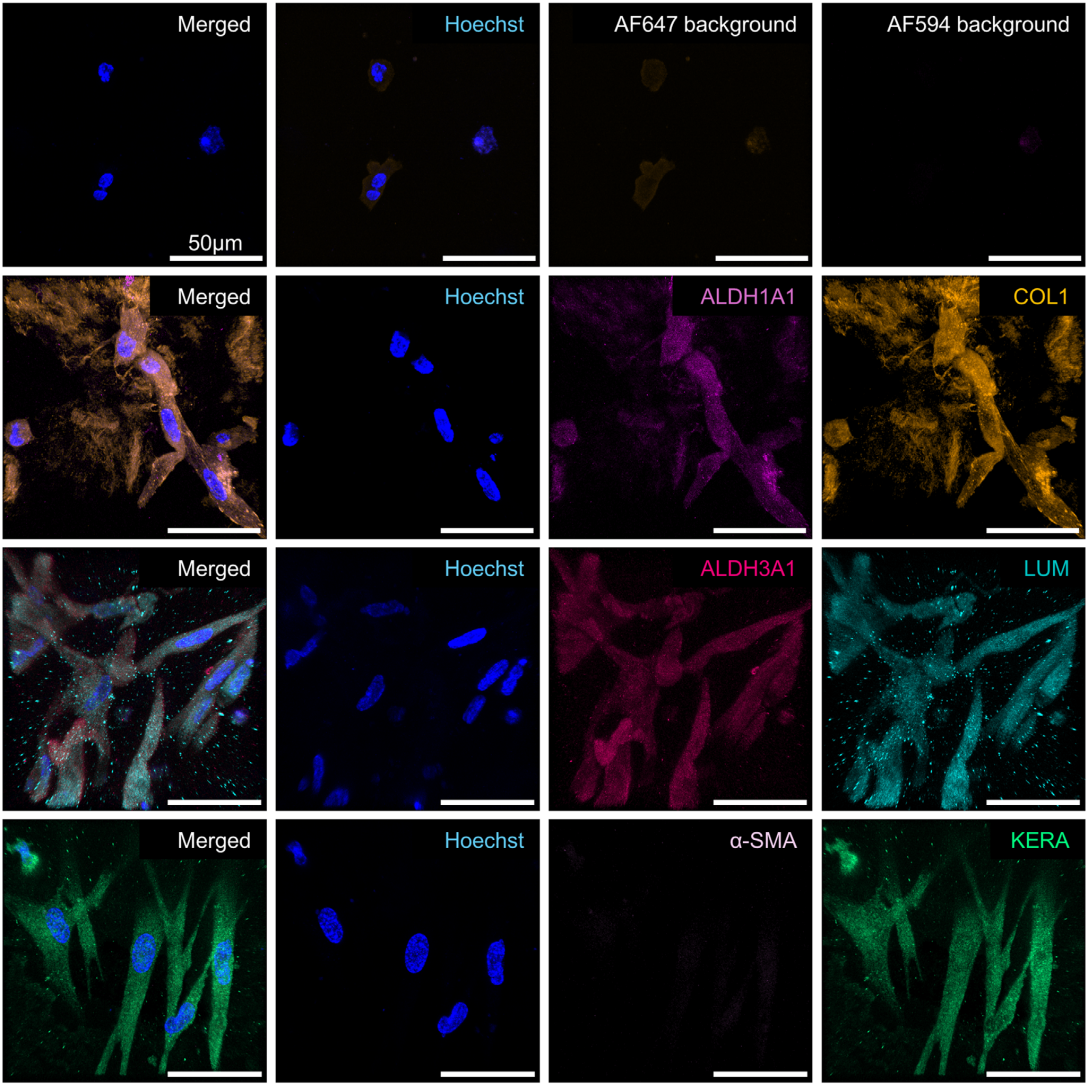
Immunofluorescence staining of 3D printed CSK‐MSC for ALDH1A1 (light pink), Collagen 1 (COL1, orange), ALDH3A1 (dark pink), Lumican (LUM, turquoise), α‐SMA (lilac), and Keratocan (KERA, green) with Hoechst Nuclear Staining (blue) and Background Controls post‐differentiation. Scale bars represent 50 µm. Representative images from samples prepared with pooled donors (*n* = 3).

Pre‐differentiation and post‐differentiation analysis in IF staining show similar results compared to the 3D differentiation. CSK‐specific markers Lumican, Keratocan, ALDH1A1, ALDH3A1 showed no signal in BM‐MSC in the bioprinted construct prior to differentiation (**Figure** **7**) and positive signal for CSK‐MSC post‐differentiation (**Figure** **8**). Collagen 1 showed a slight signal prior to differentiation and an upregulated signal postdifferentiation. Finally, α‐SMA showed no signal pre‐ and post‐differentiation, validating as well that the bioprinting process did not influence the myofibroblastic differentiation of the CSK‐MSC (Figures 7 and 8).

## 3 Discussion

This study presents a novel differentiation protocol for generating CSK‐like cells from BM‐MSC in both 2D and 3D culture systems, with significant implications for corneal tissue engineering and bioprinting applications. Gene expression analysis revealed that BM‐MSC underwent successful transcriptional changes during differentiation into CSK‐like cells in both 2D and 3D cultures. Upregulation of key CSK‐related genes, including Lumican, Keratocan, ALDH1A1, and ALDH3A1, was observed in both culture conditions, confirming the acquisition of a CSK‐like transcriptional profile.

Our approach stands out by prioritizing the evaluation of α‐SMA expression, a key marker for myofibroblast differentiation, which is often ignored in other studies investigating cornea bioprinting or MSC differentiation.^[^ ^19^, ^21^, ^22^, ^23^, ^24^ ^]^ Notably, the absence of signal in IF staining and the significantly downregulated level of expression of α‐SMA in our 3D cultures demonstrated that the differentiated cells had no myofibroblastic phenotype. These findings highlight the potential of 3D culture systems in improving CSK differentiation protocols. Future work should aim to optimize these conditions for higher scalability and explore in vivo applications to evaluate the functional integration of these cells in corneal regeneration therapies.

Importantly, the differentiation process in 3D cultures, carried out over 21 days (7 days in MSC medium, necessary to allow cell spreading, plus 14 days in differentiation medium), provided a more physiologically relevant environment that enhanced CSK marker expression, mimicking the native corneal microenvironment more closely than traditional 2D cultures. In 2D, cells reached high confluency by the end of the 14‐day differentiation period and were maintained in CSK medium to support cell viability and allow expansion prior to analysis. Although passaging is not required in the 3D constructs, an equivalent 3‐day incubation in CSK medium was applied to ensure consistent post‐differentiation handling across both models before IF and qPCR analysis.

Another advancement in this study relies in the preconditioning of BM‐MSC on collagen‐coated plates before 2D differentiation. This collagen coating, which has been previously reported to reduce α‐SMA expression in CSK culture,^[^ ^25^ ^]^ was incorporated to the culture of the BM‐MSC pre‐differentiation to lower the risk of myofibroblast differentiation, a common issue in corneal tissue engineering that leads to undesirable fibrotic outcomes.^[^ ^26^ ^]^ Although qPCR analysis did not reveal significant differences in the expression of positive CSK markers between 2D and 3D conditions, the significantly lower expression of α‐SMA in 3D CSK‐MSC indicates a reduced myofibroblastic phenotype, which we consider an indicator of successful differentiation toward a quiescent keratocyte lineage. This step is crucial because the suppression of myofibroblast characteristics is necessary for maintaining the transparency and biomechanical properties of the corneal stroma,^[^ ^27^ ^]^ making this protocol particularly suited for regenerative therapies and bioprinting applications.

The 3D differentiation parameters used in this study offer significant potential for in situ and in vivo bioprinting of the corneal stroma. The enhanced expression of ECM components such as Collagen I in the 3D culture further supports the feasibility of integrating these cells into biofabricated constructs^[^ ^28^ ^]^ or into other bioink formulations used for corneal tissue engineering.^[^ ^29^ ^]^ The ability to generate CSK‐like cells within a drop‐on‐demand 3D bioprinted hydrogel matrix that mimics the native stromal environment demonstrated that this system could be directly applied to biofabrication strategies where cells are printed into a corneal scaffold or directly onto the eye during in situ bioprinting process. The prolonged differentiation period (21 days) also aligns with the natural developmental timeline of stromal cells, which may improve the integration and functionality of biofabricated tissues in vivo.

By using the potential of BM‐MSC differentiation and optimizing the cellular microenvironment through preconditioning and 3D culture systems,^[^ ^30^ ^]^ this protocol was then adapted for bioprinting technologies aimed at reconstructing the corneal stroma. This study provides a potential cell source for autologous therapies, addressing corneal damage with patient‐specific cells to reduce immune rejection.^[^ ^31^ ^]^ In personalized medicine, BM‐MSC from individual patients can be used to develop tailored corneal treatments.

While our study demonstrates significant advancements in CSK differentiation and bioprinting, some limitations should be considered. First, our differentiation protocol successfully promoted the expression of CSK‐specific markers, but the long‐term stability of these phenotypes in vivo remains untested. Future studies should investigate whether the differentiated cells maintain their CSK‐like characteristics after implantation in a corneal environment. Future studies should focus on optimizing cell densities and patterning, gel formulations, and print fidelity to ensure precise deposition of these cells, as well as ex vivo testing to evaluate the long‐term integration and function of bioprinted corneal models in the host tissue.

## 4 Conclusion

In conclusion, the novel differentiation protocol described in this study represents a promising step forward in the field of corneal tissue engineering and bioprinting. By preconditioning BM‐MSCs to lower α‐SMA expression and utilizing a 3D culture system that supports robust CSK marker expression, this study opens new avenues for the development of biofabricated corneal constructs. Moreover, the successful 3D differentiation of BM‐MSC post‐printing provides a solid foundation for the future of in situ and in vivo bioprinting applications, with the ultimate goal of restoring vision to patients with corneal damage or disease.

## 5 Experimental Section

### Cell Culture

Human Corneal Keratocytes (HCK) were purchased from Innoprot (P10872, Innoprot, Spain) and subcultured in CSK basal medium made of Dulbecco's Minimum Essential Medium DMEM/F12 (P04‐41150, Pan Biotech, Germany) supplemented with 0.5% Fetal Bovine Serum (FBS) (P30‐3031, Pan Biotech, Germany), 1% MEM Vitamin Solution (M6895, Sigma–Aldrich, Germany), 1 % MEM Non‐Essential Amino Acids (M7145, Sigma–Aldrich, Germany), 1 mm L‐Ascorbate 2‐Phosphate (LA2P) (A8960, Merck, Germany), 10 µM ROCK‐inhibitor (688002‐M, Millipore, Germany), 10 ng mL^−1^ Recombinant Human Insulin‐like Growth Factor‐1 (CB‐1104113, Pan Biotech, Germany), and 1% Penicillin‐Streptomycin (Pen/Strep) (P4333, Sigma Aldrich, Germany).

Human BM‐MSC from 3 donors (Male 63, Female 62, Female 72, all Caucasian) were purchased from Promo Cell (C‐12974, Promo Cell, Germany), and subcultured in Mesenchymal Stromal Cell Growth Medium 2 (C‐28009, Promo Cell, Germany), and 1% Pen/Strep. Cells were seeded at a density of 5000 cells per cm^2^. Once 70% of confluency was reached, medium was substituted with keratocyte differentiation (KD) medium consisting of DMEM/F12, 1% MEM Vitamin Solution, 1% MEM Non‐Essential Amino Acids, 1% Insulin‐Transferrin‐Selen (41400045, Gibco, Germany) 1 mm LA2P, 20 ng mL^−1^ Fibroblast Growth Factor‐2 (CB‐1102021, Pan Biotech, Germany), 0.1 ng mL^−1^ Transforming Growth Factor‐β3 (78131, Stem Cell Technologies, Germany) and 1% Pen/Strep. KD medium was changed every 2 days. MSC‐derived keratocytes (CSK‐MSC) were collected for expansion on day 14 of differentiation by detaching with 0.25% trypsin‐EDTA and plated in CSK medium.

Human Stromal Fibroblasts (HSF) were kindly provided by Aachen University Clinic cornea bank and were cultivated in DMEM/F12 supplemented with 5% FBS, 1% Pen/Strep, and 1% Amphotericin B (15290018, Gibco, Germany).

Two surface conditions of T75 cell culture flasks (83.3911.002, Sarstedt, Germany) were utilized: uncoated (standard tissue culture plastic) and collagen‐coated. Collagen (type I from calf skin in HCl; L7213, Sigma–Aldrich, Germany) was applied at a density of 10 µg cm^−^^2^ by incubating each flask with 9.4 mL of an 80 µg mL^−1^ coating solution, prepared by diluting a 4 mg mL^−1^ stock solution in sterile 1X phosphate‐buffered saline (PBS). Coating was carried out for 1 h at 37 °C. BM‐MSC were considered pre‐conditioned when cultivated for at least 7 days on collagen‐coated plates. HCK were cultivated only on collagen‐coated plates and HSF were cultivated on uncoated plates.

### Hydrogel Preparation

Stock solutions of 3 wt.% low gelling temperature agarose (A4018, Sigma Aldrich, Germany) were prepared by dissolving the agarose in milli‐Q water under autoclaving conditions (121 °C for 2h).

Stock solutions of 0.3 v/v % collagen gel were prepared by mixing 80 v/v % 4 mg mL^−1^ acidic collagen solution (type I from calf skin in HCl; L7213, Sigma–Aldrich, Germany) with 10 v/v % 10X DMEM (D2429, Sigma Aldrich, Germany), 5 v/v % HEPES buffer, and neutralized with 5 v/v % 0.7 M sodium hydroxide (S5881, Sigma Aldrich, Germany).

Agarose‐collagen gels were prepared from the two stock solutions with a final concentration of 0.5 % agarose and 0.2 % collagen I.

### 3D Culture

To cultivate the cells in 3D, cells were harvested from culture plates using 0.25 % Trypsin/EDTA, counted and resuspended to reach a concentration of 10^6^ cells per mL of gel. Cells were encapsulated in the gel by gently mixing the cells into the collagen and further adding the agarose until a homogenous mixture was achieved.

50 µL of cell‐gel mixtures were plated in a 96‐well plate. After polymerization, 100 µL of medium was added to the well to cultivate the cells. Polymerization occurred within 10 min at Room Temperature (RT).

MSC and HCK cast cells were cultivated respectively in MSC medium and CSK medium. The 3D differentiation process was conducted as follows: after 7 days of 3D culture of the BM‐MSC in MSC basal medium, the medium was replaced with KD medium for 14 days, with media change conducted every two days. Finally, KD medium was substituted with CSK medium at day 21, and cells were kept in this medium for 3 days prior to immunofluorescence staining and RNA isolation. HCK were kept in 3D culture for 7 days prior to IF staining and RNA isolation.

### 3D Bioprinting

The 3D corneal model was designed with Autodesk Inventor (Autodesk, USA) with a horizontal diameter of 18 mm and a height of 18 mm resulting in a half‐sphere. In this study, bioinks were 3D printed using a drop‐on‐demand bioprinter (SuperFill with custom design, Black Drop Biodrucker GmbH, Germany) using an electromagnetic microvalve with a nozzle diameter of 300 µm (Fritz Gyger AG, Switzerland) and a pressurized air supply with a constant pressure of 0.5 bar.

The bioprinting process followed the protocol of Duarte Campos et al., 2019.^[^ ^10^ ^]^ Briefly, the BM‐MSC pooled from the 3 different donors were casted in a 0.5 % Agarose – 0.2 % Type 1 Collagen bioink with a concentration of 10^6^ cells mL^−1^ and kept at 30 °C during the printing process. In each layer, droplets were first deposited in spaced positions (red, A drops), then additional droplets were placed in between (blue, B drops) to fill the gaps. This two‐step strategy was designed to prevent early merging of the droplets before crosslinking, which can occur with low‐viscosity hydrogels (Figure 6A). The polymerization of the BM‐MSC loaded bioink took place at RT for 10 min. The hydrogels were then immerged in MSC basal medium and kept in a humidified incubator at 37 °C. After 7 days, MSC medium was substituted with KD medium for 14 days, with media change conducted every 2 days. Finally, KD medium was substituted with CSK medium at day 21, and cells were kept in this medium for 3 days prior to immunofluorescence staining.

### Immunofluorescence Staining

Cells were seeded on 96 µ‐well plates (89606, Ibidi, Germany) at a density of 5000 cells per cm^2^ for 2D cultures and were casted directly in the wells of these plates as described earlier for 3D cultures. After 48 h, samples were washed three times with PBS supplemented with 1 mM MgCl_2_, and then fixed for 10 min in 4 % Paraformaldehyde (47347.9M, VWR, Germany). After fixation, samples were washed again three times with PBS and permeabilized by 0.2 % Triton X‐100 (1086031000, VWR, Germany) for 10 min at RT. Samples were washed again three times with PBS and were blocked 1 h with 5 % Donkey Serum (P30‐0101, Pan Biotech, Germany) and 2 h with 20 % Donkey Serum respectively for 2D and 3D cultures. After blocking, samples were then incubated with the first antibody solution diluted in the blocking solution, as described in **Table** **1**. Primary antibody concentrations were doubled for 3D cultures. Primary antibody incubation was followed by washing three times with PBS containing 0.2 % Tween buffer in the dark. Positive immunoreaction of the primary antibody was then detected by secondary antibodies conjugated with a specific fluorophore, namely goat‐anti‐rabbit (AF594) and goat‐anti‐mouse (AF647), for 1 h at RT in the dark. Nucleic acids staining was performed alongside secondary antibody staining with Hoechst. Fluorescence images were obtained by confocal microscopy (LSM780, ZEISS, Germany) and as z‐stack for 3D samples with a range of 15–20 µm. Post‐processing was performed with IMARIS (10.1.1, Oxford Instruments, UK) by applying background subtraction and deconvolution.

**Table 1:** Antibody list and dilution factors.

Primary antibody		
Target antigen	Host	Dilution factor
α‐SMA (MA5‐11547, Thermo Fisher, Germany)	Mouse	1:500 (0.8 µg mL^−1^)
ALDH1A1 (60171‐1‐Ig, Proteintech, Germany)	Mouse	1:500 (2 µg mL^−1^)
ALDH3A1 (68036‐1‐Ig, Proteintech, Germany)	Mouse	1:400 (2.5 µg mL^−1^)
Keratocan (bs‐11054R, Thermo Fisher, Germany)	Rabbit	1:200 (5 µg mL^−1^)
Lumican (bs‐5890R, Thermo Fisher, Germany)	Rabbit	1:200 (5 µg mL^−1^)
Collagen I (14695‐1‐AP, Proteintech, Germany)	Rabbit	1:400 (1.5 µg mL^−1^)

Secondary antibody		
Fluorophore	Reactivity	Dilution factor
Alexa Fluor 594 (ab150080, abcam, Germany)	Rabbit	1:400 (2µg mL^−1^)
Alexa Fluor 647 (ab150115, abcam, Germany)	Mouse	1:500 (2 µg mL^−1^)
Hoechst (62249, Fisher scientific, Germany)		1:500 (1 µg mL^−1^)

### qPCR Analysis

Cells grown in a 2D culture were prepared for the isolation by washing with PBS twice and pelleting at 12 000 × g. The pellet was snap frozen by placing it in liquid nitrogen. The RNA isolation was carried out with an RNA isolation kit (NucleoSpin RNA Plus, 740984.50, Macherey‐Nagel, Germany). RNA isolation from a 3D cell culture was performed following the phenol‐chloroform method. Briefly, samples were homogenized with 1 ml TRIzol (15596026, Thermo Fisher, Germany) and snap frozen in liquid nitrogen after digestion. The thawed sample was mixed with 200 µl chloroform, incubated for 2 min, and centrifuged for 15 min at 12 000 × g at 4 °C. Upon centrifugation, the sample was divided into the phenol‐chloroform phase, the interphase, and the upper aqueous phase. The latter that contains the RNA was transferred to a new tube, and it was precipitated by adding 500 µl isopropanol for 10 min. The precipitate was then centrifuged for 10 min at 12 000 × g. The supernatant was discarded, and the RNA pellet was washed in 1 mL of 75% ethanol. Then, the sample was centrifuged for 15 min at 12 000 × g, and the supernatant was discarded. After airdrying the RNA pellet, it was solubilized in 10 µl RNA‐free water.

cDNA synthesis was perform using a synthesis kit (RevertAid First Strand cDNA Synthesis). Gene expression was quantified by qRT‐PCR reactions, performed on 10 µg cDNA with the Sso Advanced Universal SYBR Green Supermix (1725270 Bio‐Rad, US) and the Bio‐Rad CFX Opus 384 Thermocycler.

Fold change was calculated by the 2^−ΔΔCt^ Method.

(1) $$\Delta Ct=Ct\left(Target\right)-Ct\left(Reference\right)$$

(2) $$\Delta \Delta Ct=\Delta Ct\left(Sample\right)-\Delta Ct\left(Control\right)$$

(3) $$Fold\;change=2^{-\Delta \Delta Ct}$$

Glyceraldehyde 3‐phosphate dehydrogenase (GAPDH) was used as housekeeping gene.

The range of the fold change was then calculated based on the standard deviations (s) from the Ct values.

(4) $$2^{-\left(\Delta \Delta \mathrm{Ct}\pm s\right)},\mathrm{with}s=\sqrt{{\left(S_{\mathrm{target}}\right)}^{2}-{\left(S_{\mathrm{referebce}}\right)}^{2}}$$

The level of expression of CSK markers before and after differentiation both in 2D and 3D cultures were compared to the level of expression of these markers in BM‐MSC cultivated in MSC medium respectively for 2D and 3D cultures. The level of expression was normalized to BM‐MSC prior to differentiation in 2D cultures. Forward and reverse primers used for qPCR analysis are summarized in **Table** **2**.

**Table 2:** qPCR primers.

Target	Forward primer	Reverse primer
α‐SMA	CCCTTGAGAAGAGTTACGAG	CAGACTCCATCCCGATGAA
ALDH1A1	AAAGCCATAACAATCTCCTCTG	TACTCTCCCAGTTCTCTTCC
ALDH3A1	CATTGGCACCTGGAACTACC	GGCTTGAGGACCACTGAGTT
Keratocan	GAAAAAGGAGCCCTAAGCCA	CTCCAGATTGCTAAAGGTCCCT
Lumican	CAAGACAGTAAGGATTCAAACC	TACCACCAATCAATGCCAG
GAPDH	GTCAAGGCTGAGAACGGGAA	CTTTTGGCTCCCCCCTGCAAAT

### Statistical Analysis

All results were presented as mean ± standard deviation (SD) with the corresponding sample size described in the respective figure captions. Comparisons between multiple experimental groups with two factors were conducted using an ordinary one‐way ANOVA with Tukey's post hoc test (Prism 10.1.2, GraphPad Software, Boston, USA). Statistical significance was labeled as *p < 0.05, **p < 0.01 and ***p < 0.001.

## Conflict of Interest

The authors declare no conflict of interest.

## Supporting information



Supporting Information

## Data Availability

The data that support the findings of this study are available from the corresponding author upon reasonable request.
