# Supplementary material for: 3D Differentiation of Bone‐Marrow Derived Mesenchymal Stromal Cells into the Keratocyte Lineage for Corneal Bioprinting
Source: Adv Healthc Mater. 2025 Jul 29;14(28):2405073. doi: 10.1002/adhm.202405073 (PMC12581885; doi:10.1002/adhm.202405073)
Supplement: Supplementary file 1 — Supporting Information [file ADHM-14-0-s001.docx]

**Supplementary section**

**Three-dimensional differentiation of bone-marrow derived mesenchymal stromal cells into the keratocyte lineage for corneal bioprinting**

Alexandre Taoum^1,^*, Ronja Friede^1^, Ole Thaden^1^, Oleksandr Rachynskyi^1^, Andrea Frank^1^, Meng Wang^1^, Friederike Dehli^1^, Matthias Fuest^2^, Daniela Duarte Campos^1,^*

^1^ Bioprinting & Tissue Engineering Group, Center for Molecular Cell Biology (ZMBH), Heidelberg University

^2^ Ophthalmology Clinic, RWTH Aachen University Hospital

*Correspondence to: [a.taoum@zmbh.uni-heidelberg.de](mailto:a.taoum@zmbh.uni-heidelberg.de); [dcampos@uni-heidelberg.de](mailto:dcampos@uni-heidelberg.de)


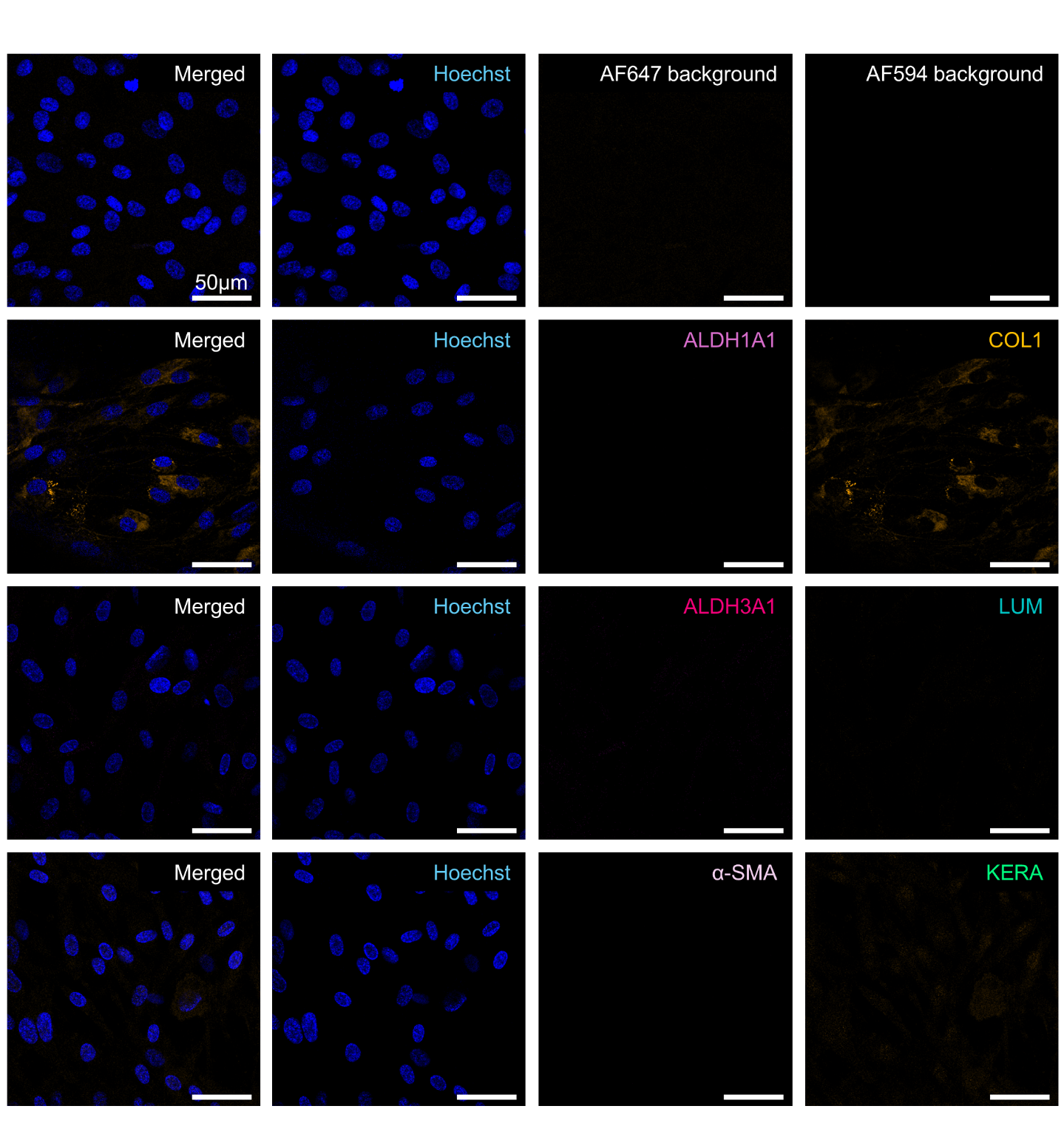


**Figure S1**: Immunofluorescence staining of 2D BM-MSC for ALDH1A1 (light pink), Collagen 1 (COL1, orange), ALDH3A1 (dark pink), Lumican (LUM, turquoise), α-SMA (lilac), and Keratocan (KERA, green) with Hoechst Nuclear Staining (blue) and Background Controls prior to differentiation. Scale bars represent 50 µm. Representative images from samples prepared with donor Female 72.

**
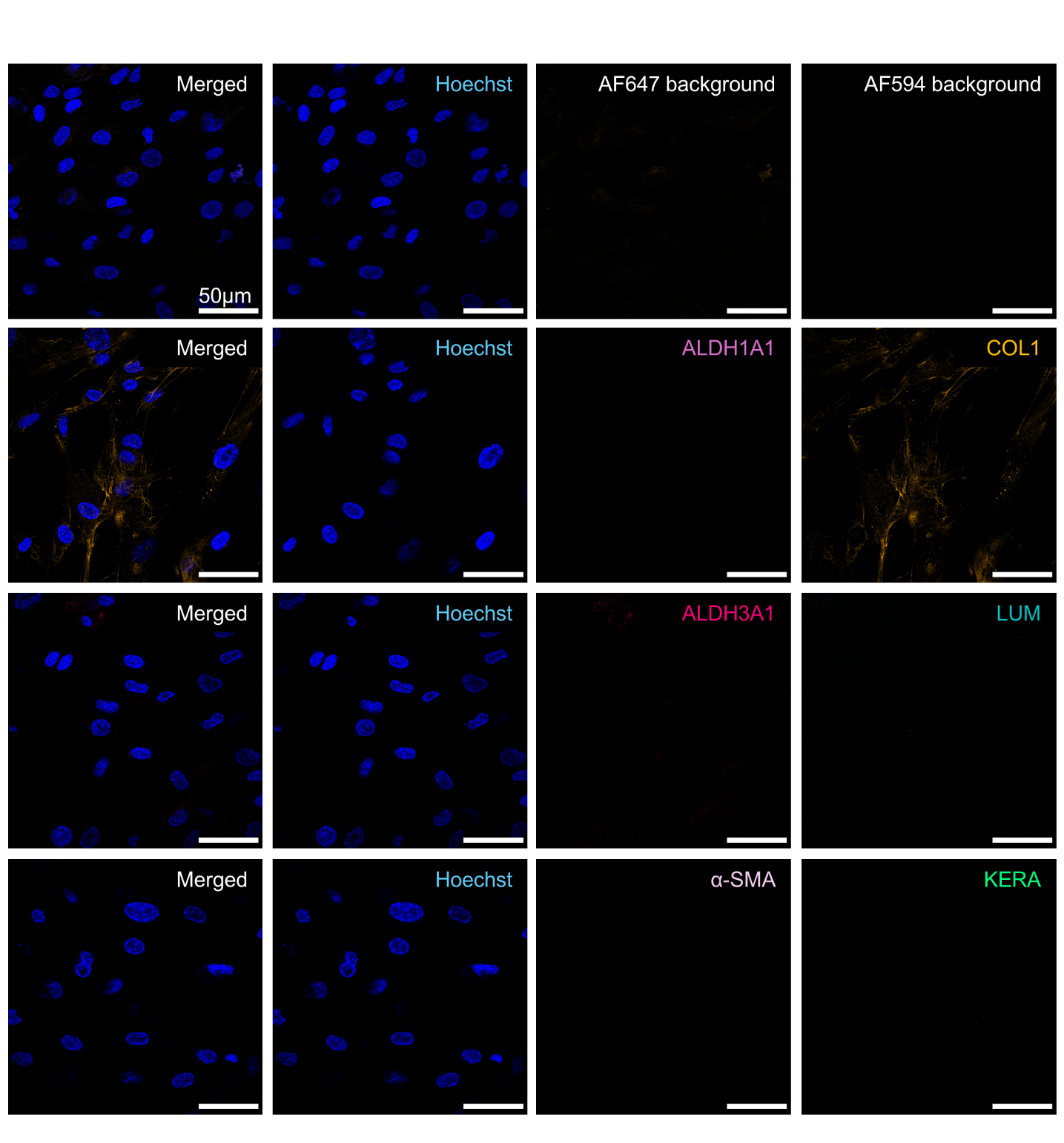
Figure S2**: Immunofluorescence staining of 2D BM-MSC for ALDH1A1 (light pink), Collagen 1 (COL1, orange), ALDH3A1 (dark pink), Lumican (LUM, turquoise), α-SMA (lilac), and Keratocan (KERA, green) with Hoechst Nuclear Staining (blue) and Background Controls prior to differentiation. Scale bars represent 50 µm. Representative images from samples prepared with donor Female 62.


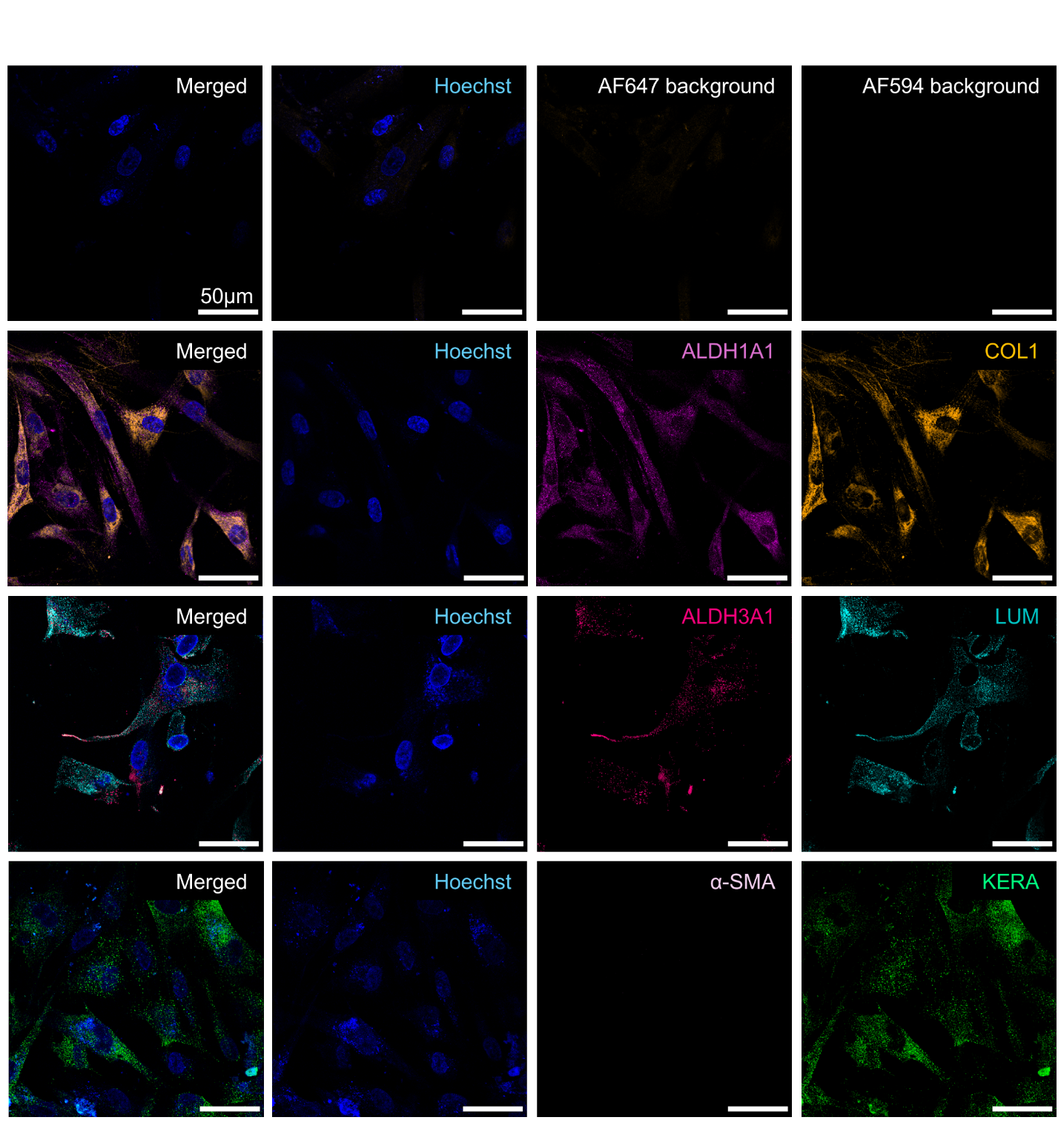
**Figure S3**: Immunofluorescence staining of 2D CSK-MSC for ALDH1A1 (light pink), Collagen 1 (COL1, orange), ALDH3A1 (dark pink), Lumican (LUM, turquoise), α-SMA (lilac), and Keratocan (KERA, green) with Hoechst Nuclear Staining (blue) and Background Controls post-differentiation. Scale bars represent 50 µm. Representative images from samples prepared with donor Female 72.


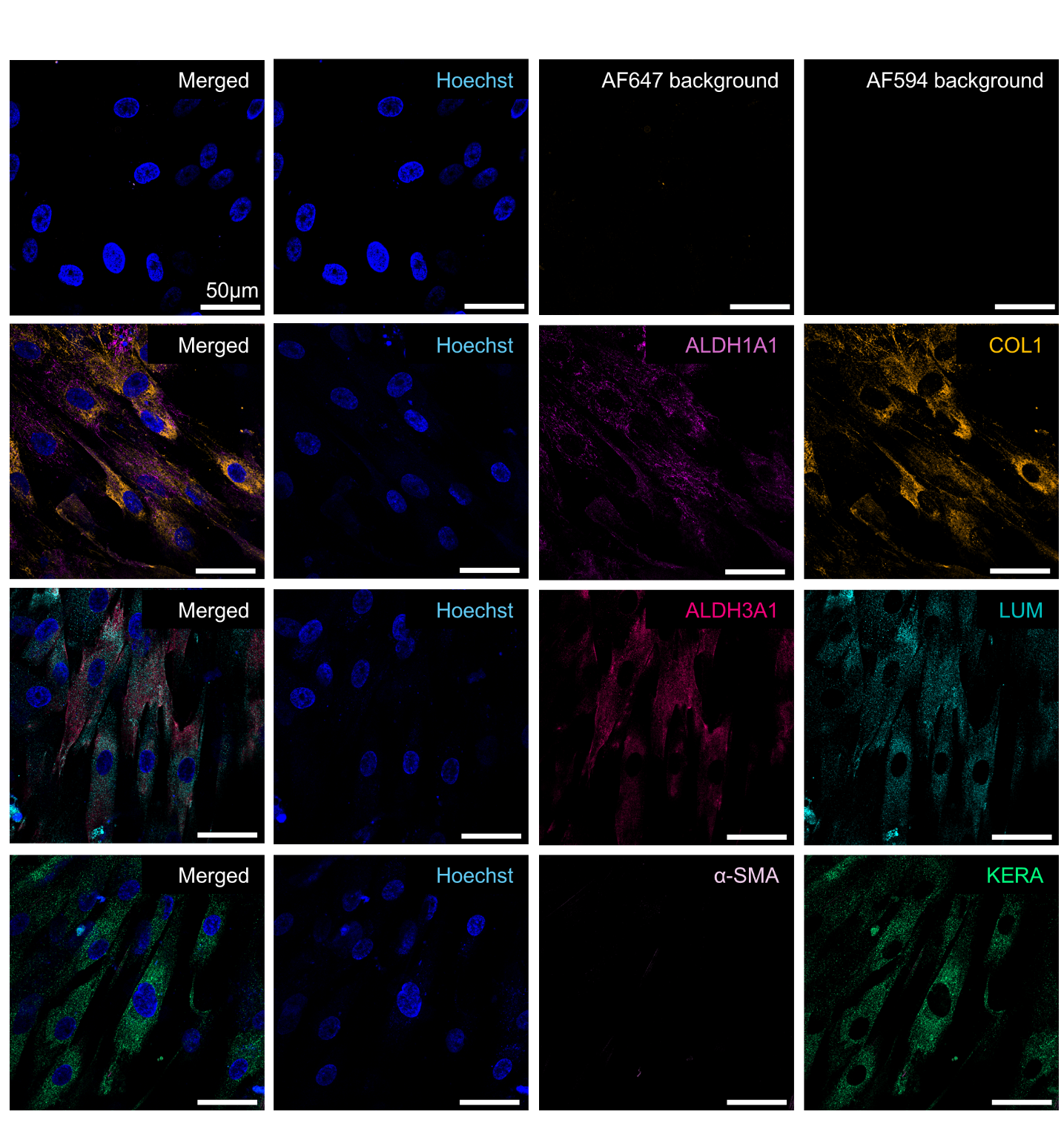
**Figure S4**: Immunofluorescence staining of 2D CSK-MSC for ALDH1A1 (light pink), Collagen 1 (COL1, orange), ALDH3A1 (dark pink), Lumican (LUM, turquoise), α-SMA (lilac), and Keratocan (KERA, green) with Hoechst Nuclear Staining (blue) and Background Controls post-differentiation. Scale bars represent 50 µm. Representative images from samples prepared with donor Female 62.

**
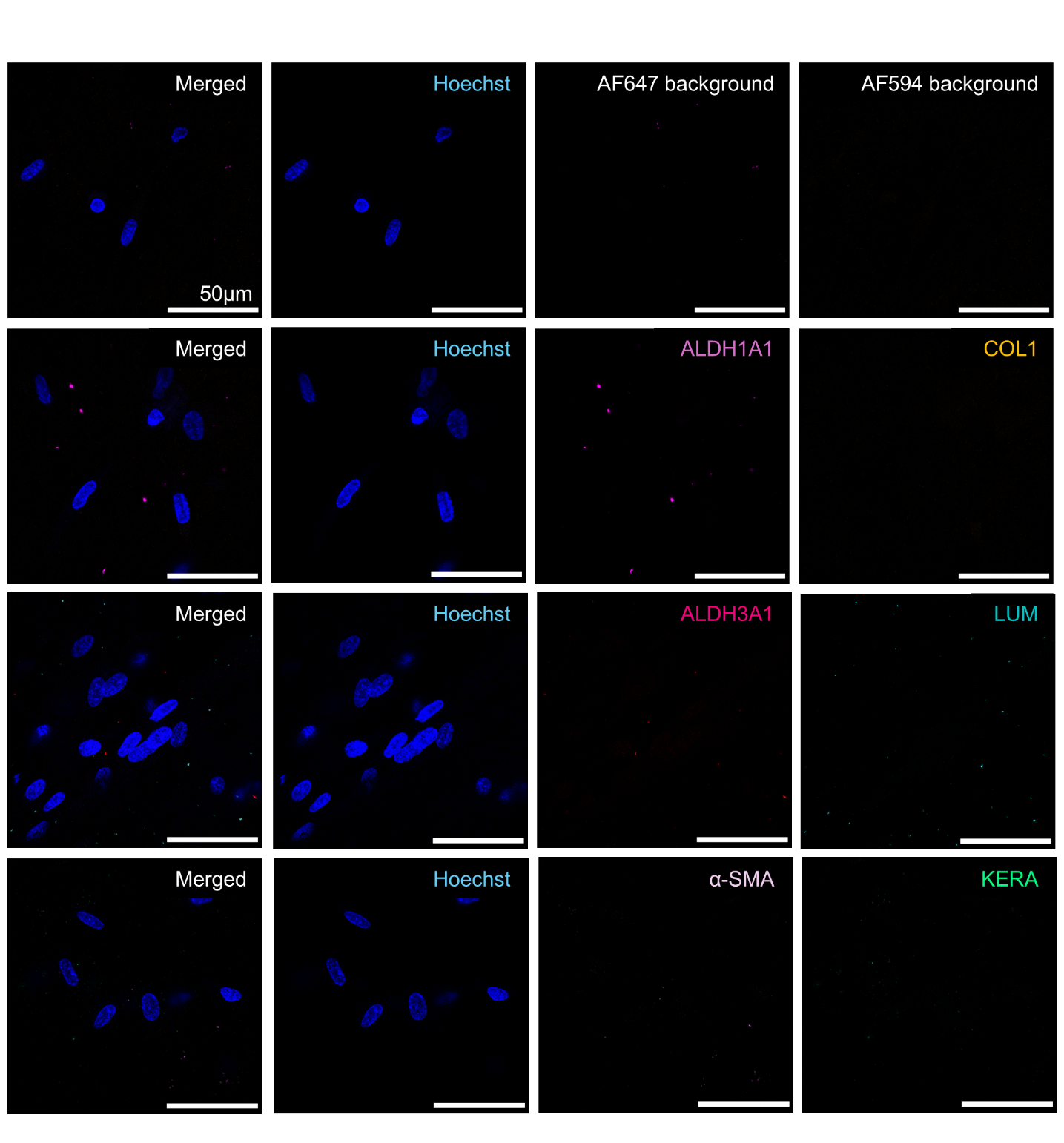
**

**Figure S5**: Immunofluorescence staining of 3D BM-MSC for ALDH1A1 (light pink), Collagen 1 (COL1, orange), ALDH3A1 (dark pink), Lumican (LUM, turquoise), α-SMA (lilac), and Keratocan (KERA, green) with Hoechst Nuclear Staining (blue) and Background Controls prior to differentiation. Scale bars represent 50 µm. Representative images from samples prepared with donor Male 63.


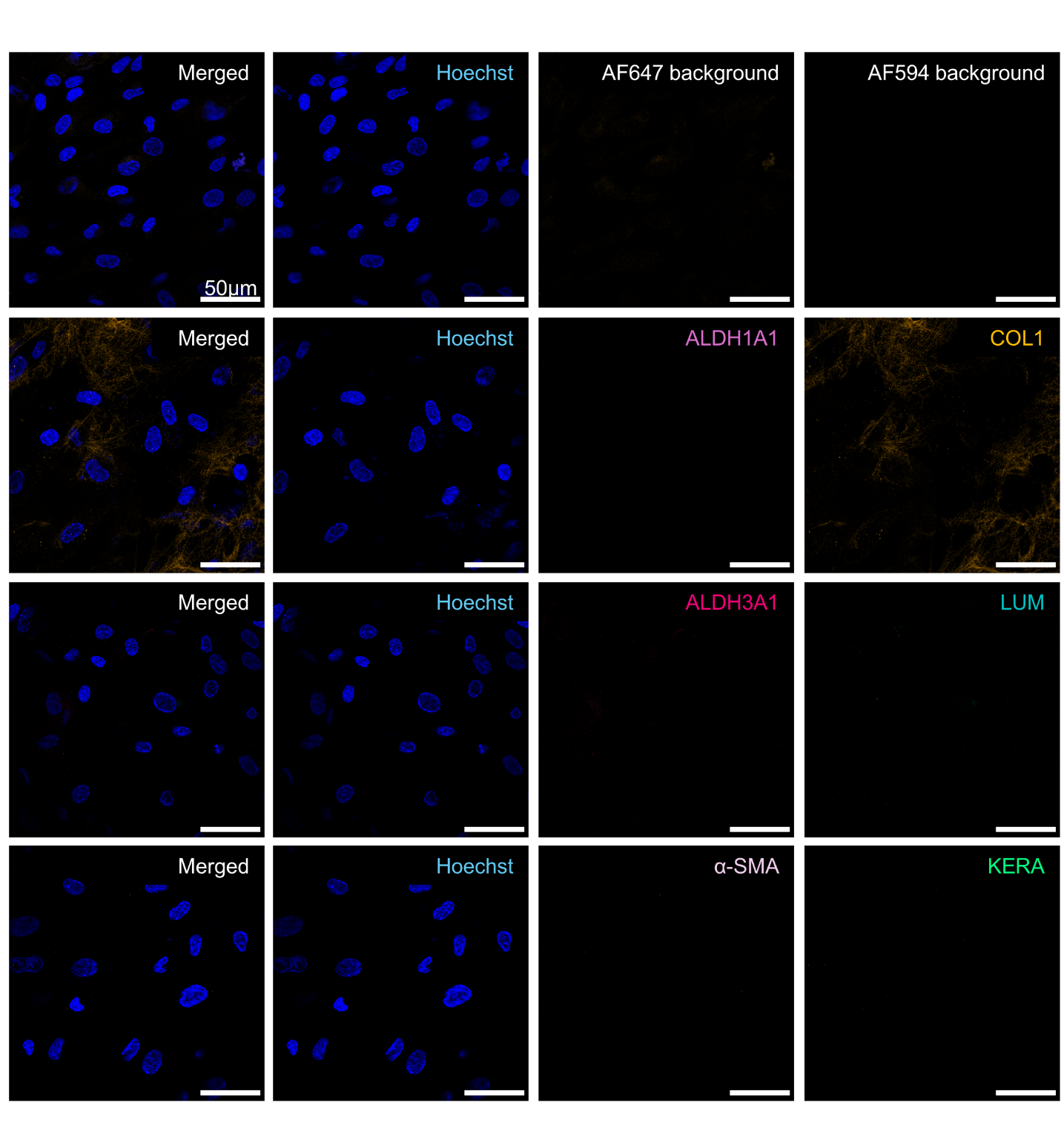


**Figure S6**: Immunofluorescence staining of 3D BM-MSC for ALDH1A1 (light pink), Collagen 1 (COL1, orange), ALDH3A1 (dark pink), Lumican (LUM, turquoise), α-SMA (lilac), and Keratocan (KERA, green) with Hoechst Nuclear Staining (blue) and Background Controls prior to differentiation. Scale bars represent 50 µm. Representative images from samples prepared with donor Female 72.


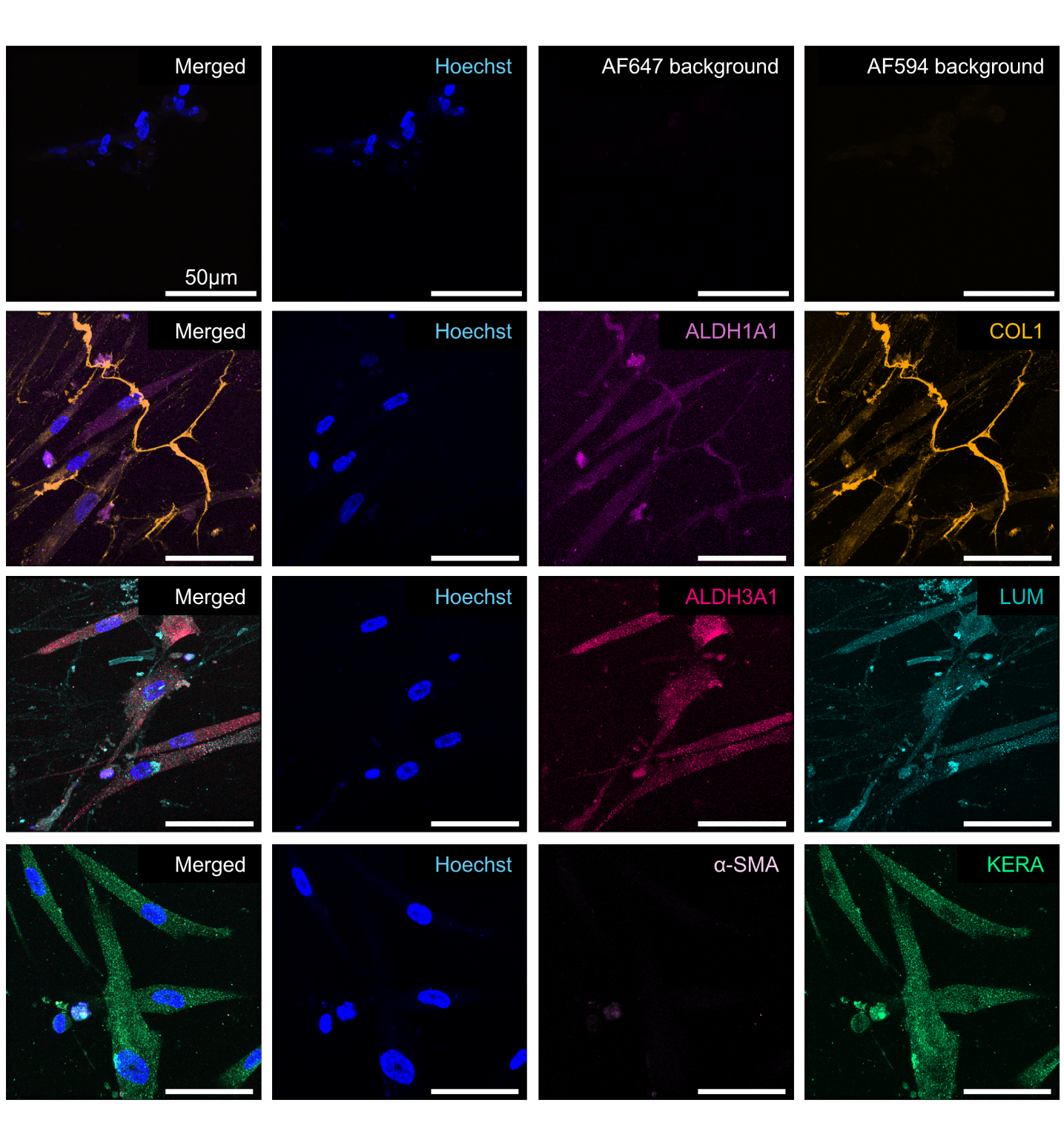


**Figure S7**: Immunofluorescence staining of 3D CSK-MSC for ALDH1A1 (light pink), Collagen 1 (COL1, orange), ALDH3A1 (dark pink), Lumican (LUM, turquoise), α-SMA (lilac), and Keratocan (KERA, green) with Hoechst Nuclear Staining (blue) and Background Controls post-differentiation. Scale bars represent 50 µm. Representative images from samples prepared with donor Male 63.


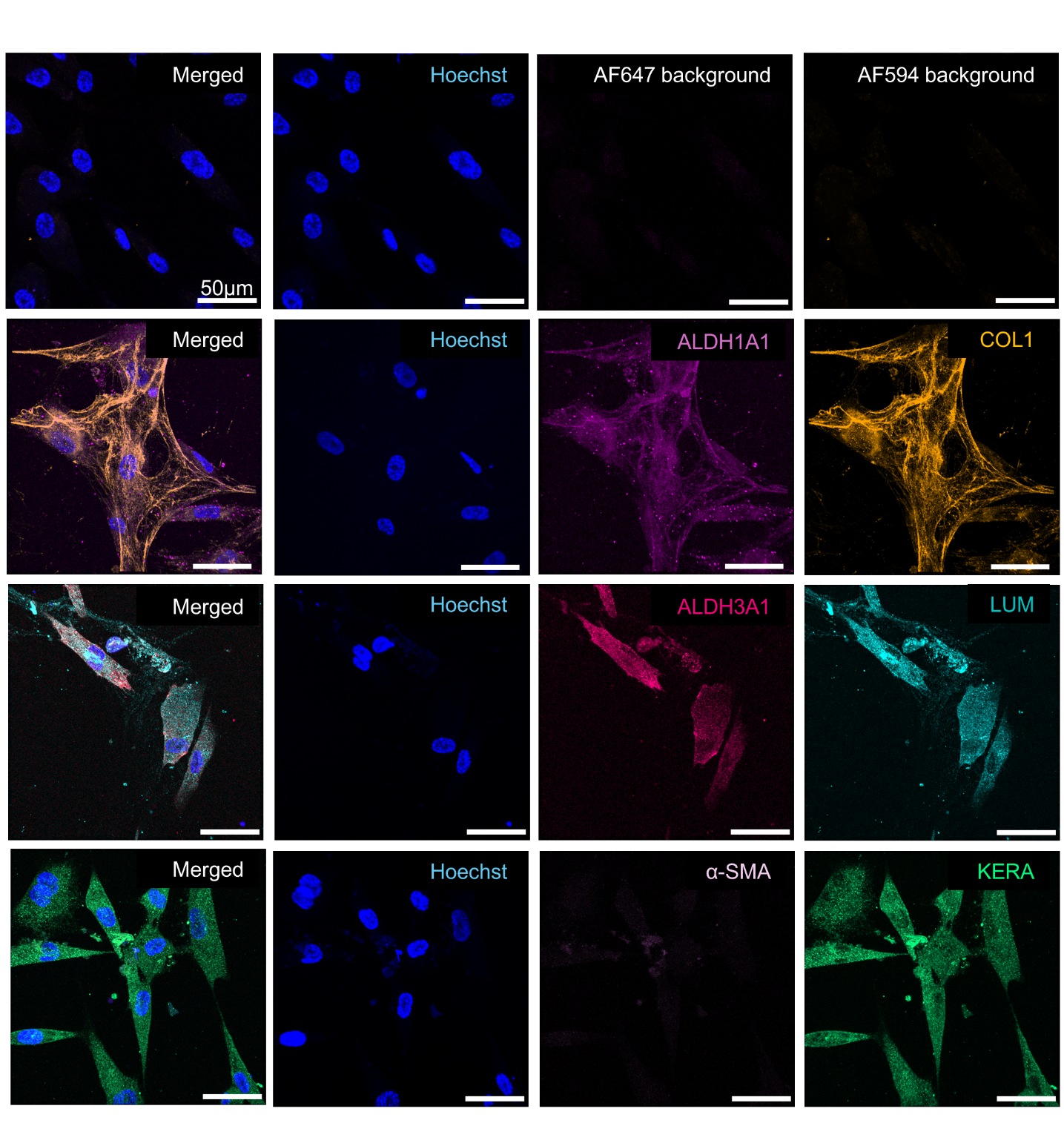


**Figure S8**: Immunofluorescence staining of 3D CSK-MSC for ALDH1A1 (light pink), Collagen 1 (COL1, orange), ALDH3A1 (dark pink), Lumican (LUM, turquoise), α-SMA (lilac), and Keratocan (KERA, green) with Hoechst Nuclear Staining (blue) and Background Controls post-differentiation. Scale bars represent 50 µm. Representative images from samples prepared with donor Female 63.


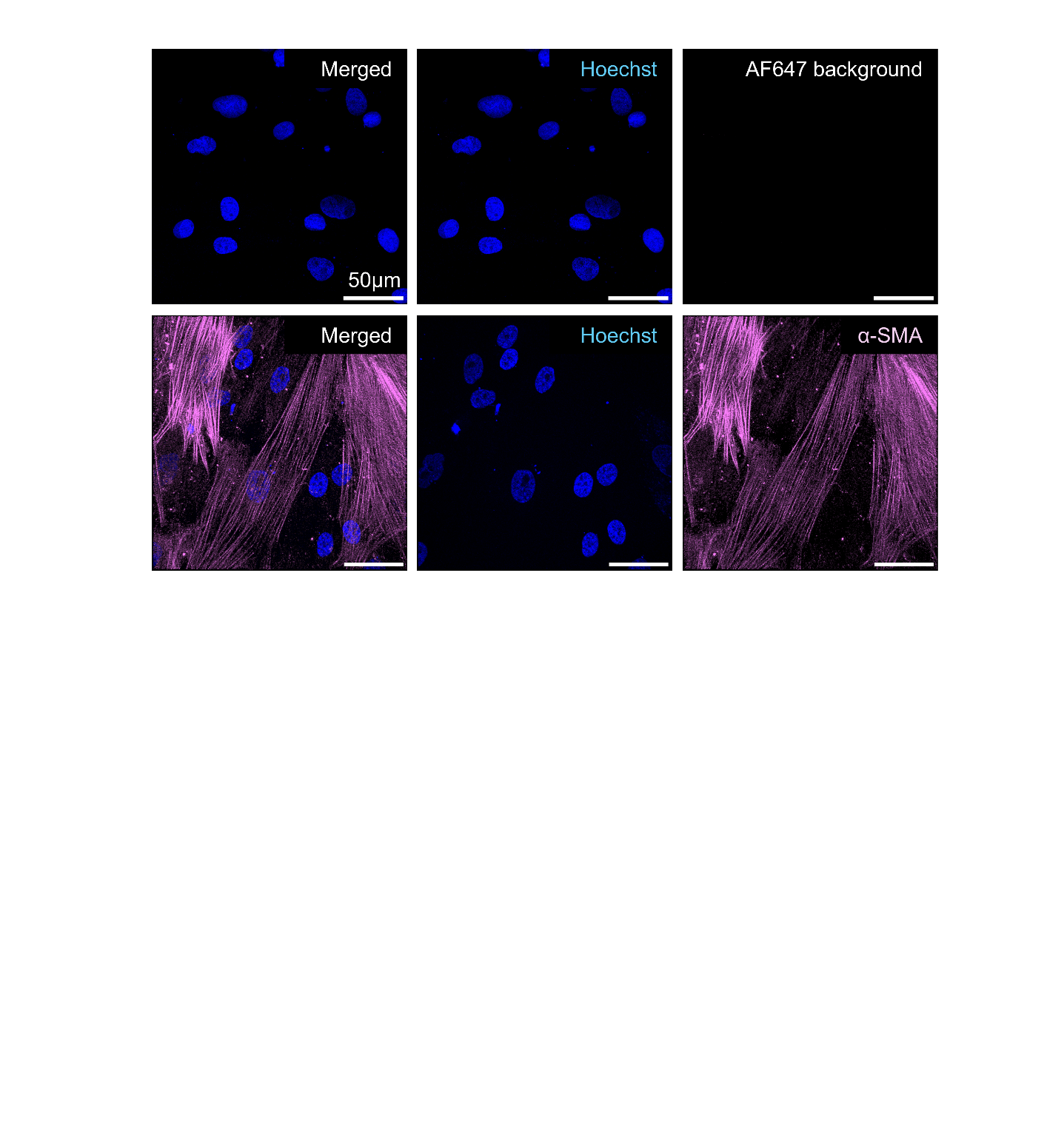


**Figure S9**: Immunofluorescence staining of 2D HSF for α-SMA (lilac) with Hoechst Nuclear Staining (blue) and Background Control. Scale bars represent 50 µm.
